# Decreasing methylation of pectin caused by nitric oxide leads to higher aluminium binding in cell walls and greater aluminium sensitivity of wheat roots

**DOI:** 10.1093/jxb/erv514

**Published:** 2015-12-09

**Authors:** Chengliang Sun, Lingli Lu, Yan Yu, Lijuan Liu, Yan Hu, Yiquan Ye, Chongwei Jin, Xianyong Lin

**Affiliations:** ^1^MOE Key Laboratory of Environment Remediation and Ecological Health, College of Natural Resource & Environmental Sciences, Zhejiang University, Hangzhou 310058, PR China; ^2^Key Laboratory of Subtropical Soil Science and Plant Nutrition of Zhejiang Province, College of Environmental and Resource Sciences, Zhejiang University, Hangzhou 310058, PR China

**Keywords:** Aluminium, cell wall, nitric oxide, pectin, pectin methylation, pectin methylesterase.

## Abstract

Nitric oxide (NO) is an important bioactive molecule involved in cell wall metabolism, which has been recognized as a major target of aluminium (Al) toxicity. We have investigated the effects of Al-induced NO production on cell wall composition and the subsequent Al-binding capacity in roots of an Al-sensitive cultivar of wheat (*Triticum aestivum* L. cv. Yang-5). We found that Al exposure induced NO accumulation in the root tips. Eliminating NO production with an NO scavenger (cPTIO) significantly alleviated the Al-induced inhibition of root growth and thus reduced Al accumulation. Elimination of NO, however, did not significantly affect malate efflux or rhizosphere pH changes under Al exposure. Levels of cell wall polysaccharides (pectin, hemicelluloses 1, and hemicelluloses 2) and pectin methylesterase activity, as well as pectin demethylation in the root apex, signiﬁcantly increased under Al treatment. Exogenous cPTIO application significantly decreased pectin methylesterase activity and increased the degree of methylation of pectin in the root cell wall, thus decreasing the Al-binding capacity of pectin. These results suggest that the Al-induced enhanced production of NO decreases cell wall pectin methylation, thus increasing the Al-binding capacity of pectin and negatively regulating Al tolerance in wheat.

## Highlight

Aluminium-induced nitric oxide production enhances the aluminium sensitivity of wheat by decreasing pectin methylation of root cell-wall pectin, resulting in greater aluminium binding in root cell walls.

Received 12 June 2015; Revised 16 October 2015; Accepted 2 November 2015

## Introduction

Aluminium (Al) toxicity is a major factor limiting crop productivity in acidic soils, which account for around 30% of the world’s arable land and approximately 50% of the world’s potentially arable land (Kochian *et al.*, 2004; Ma, 2007; Kochian *et al.*, 2015). Furthermore, up to 60% of the acidic soils in the world are in developing countries, where food production is critical (Kochian *et al.*, 2005; Liu *et al.*, 2014). Although it has been shown that the strong binding affinity of Al to cell components can alter a series of physiological and biochemical processes, disrupt cytoskeleton dynamics, destruct plasma membrane integrity, and distort calcium-dependent signal cascades, the underlying physiological and molecular mechanisms of Al-induced root growth inhibition are still not well understood (Kochian, 1995; Matsumoto, 2000; Rengel and Zhang, 2003; Ma, 2007).

Accumulating evidence suggests that the cell wall plays pivotal roles in the perception and manifestation of Al toxicity in plants (Horst *et al.*, 2010; Kochian *et al.*, 2015). The cell wall is the first point of contact when plant roots are exposed to Al, and serves as a major pool for the metal. For instance, approximately 85% of the Al taken up by *Zea mays* (maize) roots accumulated in the cell wall (Wang *et al.*, 2004), and more than 77% of total Al was located in the cell wall of root apices in *Triticum aestivum* (wheat; Ma *et al.*, 2004). Al bound to the cell wall negatively affects wall structure and function by increasing the rigidity and reducing cell expansion and mechanical extensibility, thus inhibiting root elongation (Van *et al.*, 1994; Tabuchi and Matsumoto, 2001; Ma *et al.*, 2004; Yang *et al.*, 2010). The major Al binding site in cell walls is generally the pectic polysaccharides, because their negatively charged carboxylic groups have a high affinity for Al (Chang *et al.*, 1999; Schmohl and Horst, 2000). Al binds preferentially to unmethylated pectin, catalysed by the activity of pectin methylesterase (PME). Recent studies suggest that cell wall hemicellulose metabolism is also susceptible to Al stress (Zhu *et al.*, 2012; Zhu *et al.*, 2014). Although the function and alteration of cell wall polysaccharides in Al-stressed roots of different plant species have been well documented (Eticha *et al.*, 2005; Horst *et al.*, 2010), the signals involved in the regulatory cascade leading to the modification of cell wall polysaccharide composition are still not well understood.

Nitric oxide (NO), a redox-active signalling molecule, is an important endogenous signalling molecule in regulating synthesis of the cell wall (Correa-Aragunde *et al.*, 2008; Xiong *et al.*, 2009; Zhang *et al.*, 2011; Ye *et al.*, 2015). For example, NO affected the cellulose content in roots of *Solanum lycopersicum* (tomato) in a dose-dependent manner (Correa-Aragunde *et al.*, 2008), and Xiong *et al.* (2009) found that an exogenous supply of NO increased the pectin and hemicellulose contents of root cell walls in *Oryza sativa* (rice). The responses and adaptations of plants to the stress of metals, including Al, have previously been associated with NO (Illéš *et al.*, 2006; Tian *et al.*, 2007; Xu *et al.*, 2010; González *et al.*, 2012; Leterrier *et al.*, 2012; Sun *et al.*, 2014). Several studies have found that disturbing the homeostasis of endogenous NO interferes with physiological processes preventing Al from entering the roots (Wang and Yang, 2005; Wang *et al.*, 2010; Zhou *et al.*, 2012). However, the corresponding physiological roles and molecular mechanisms of NO in increasing or decreasing Al accumulation in the root apex under Al toxicity remain elusive. Considering the role of the cell wall in Al toxicity and Al absorption, it is possible that NO may interfere with cell wall properties to affect their capacity to bind with Al. Here, we have investigated the effects of Al-induced NO production on cell wall composition and the subsequent Al-binding capacity of the cell wall in roots of wheat.

## Materials and methods

### Plant materials and treatment

Seeds of wheat (*Triticum aestivum* L. cv. Yang-5) were surface-sterilized with 1% (v/v) NaClO solution for 20min, and then rinsed thoroughly with deionized water. The seeds were germinated in the dark before being grown in 2.5L of 0.5mM CaCl_2_ solution (pH 4.3) in a growth chamber under a 12h/25°C day and 12h/22°C night regime, with a light intensity of 300 μmol m^−2^ s^−1^, and a relative humidity of 70%. The solution was renewed daily.

After 3 days of pre-treatment, uniform seedlings were transferred to 0.5mM CaCl_2_ (pH 4.3) that contained either 30 μM or 0 μM AlCl_3_ for another 24h. For experiments with NO scavenger treatment, 3-day-old seedlings were placed in a 0.5mM CaCl_2_ (pH 4.3) solution with 30 μM AlCl_3_ spiked with 30 μM 2-(4-carboxyphenyl)-4,4,5,5-tetramethyl-imidazoline-1-oxyl-3-oxide (cPTIO) for 24h. The concentration used in this study was based on preliminary experiments from which the maximum induced responses were obtained.

### Evaluation of Al resistance in wheat

Root elongation and plasma membrane integrity in wheat roots were determined after 24 h of treatment. Root length was measured before and after treatments. Relative root elongation was calculated as the percentage elongation of the root under the various treatments as compared with the Al-free control. The plasma membrane integrity was evaluated using Evans Blue uptake (Yamamoto *et al.*, 2001).

### Determination of NO content

The endogenous levels of NO in roots were visualized using the fluorescent probe diaminofluorescein-FM diacetate (DAF-FM DA) and epifluorescence microscopy (Sun *et al.*, 2014; Xu *et al.*, 2015). Briefly, root tips (0–10mm) were loaded with 10 μM DAF-FM DA in 20mM HEPES-NaOH buffer (pH 7.4) for 20min, washed three times with fresh buffer, and observed under an epifluorescence microscope. Fluorescence intensity was measured with the open source software Image-J (http://rsb.info.nih.gov/ij/). NO production was expressed as root fluorescence density.

### Collection of root exudates and organic acid assays

After treatment, root exudates were collected and purified according to Zheng *et al.* (2005). Briefly, collected exudates were first passed through a cation exchange column filled with 5g of Amberlite IR-120B (H^+^ form) resin, and then through an anion exchange column filled with 2g of Dowex 1X8 resin (100–200 mesh, formate form). Organic acids retained on the anion exchange resin were eluted with 15mL of 1M HCl, and the eluent was concentrated to dryness using a rotary evaporator at 40°C. The residue was re-dissolved in 1mL Milli-Q water and filtered (0.2 μm) before analysis. The concentration of malate was analysed by HPLC (Agilent 1100, USA). The mobile phase was 0.5% KH_2_PO_4_ (pH 2.0) at a flow rate of 1mL min^−1^ and the detection wavelength was at 220nm.

### Scanning electron microscope-energy dispersive X-ray microanalysis

Six root apexes (0–10mm) were excised and fixed in 2.5% (v/v) glutaraldehyde in 0.2M sodium phosphate (NaH_2_PO_4_/Na_2_HPO_4_) buffer (pH 7.2) overnight, and post-ﬁxed in 1% (w/v) OsO_4_ for 2h. The specimen was dehydrated in a graded ethanol series (30–100%; v/v), followed by a mixture of alcohol and isoamyl acetate (v:v = 1:1) for about 30min, and then transferred to pure isoamyl acetate overnight. The sample was dried in a Hitachi Model HCP-2 critical point dryer with liquid CO_2_. Root samples were observed under a Hitachi S-3400 SEM with an energy-dispersive X-ray spectrometer (EDS).

### Cell wall extraction and polysaccharide measurement

Cell wall materials were extracted according to Yang *et al.* (2011). Frozen root apexes (0–10mm) samples were thoroughly homogenized with 75% ethanol. The homogenate was kept undisturbed in ice-water for 20min. The homogenate was then centrifuged at 8000g for 10min at 4°C, and the pellets were washed for 20min each with acetone, methanol:chloroform mixture (1:1, v/v), and methanol. The supernatant was discarded and the pellet was freeze-dried.

Cell wall materials were fractionated into three fractions: pectin, hemicellulose 1 (HC1), and hemicellulose 2 (HC2). The pectin fraction was extracted twice by 0.5% (NH_4_)_2_C_2_O (ammonium oxalate) buffer containing 0.1% NaBH_4_ (pH 4) in a boiling water bath for 1h. Pellets were subsequently subjected to triple extractions with 4% KOH containing 0.1% NaBH_4_ at room temperature for a total of 24h, followed by extraction with 24% KOH containing 0.1% NaBH_4_. The pooled supernatants from the 4% and 24% KOH extractions thus yielded the HC1 and HC2 fractions, respectively. The uronic acid content in each cell wall fraction was assayed. Galacturonic acid (GalA) was used as a calibration standard and the root pectin, HC1, and HC2 contents were expressed as GalA equivalents.

### Al content measurement

Total Al content in root apexes (0–10mm) was analysed according to Osawa and Matsumoto (2001). Briefly, excised root apices were digested with 10mL of 2M HCl. The samples were digested for at least 24h with occasional shaking.

The apoplastic and symplastic Al fractions in the root tips were collected according to the method described by Yu *et al.* (1999) and modified by Wang *et al.* (2004). Briefly, freshly excised 1-cm root tips from 20 seedlings were arranged in a ﬁlter unit (Ultrafree-MC, 0.45 μm; Millipore, Bedford, MA, USA) with the cut ends facing down. The water free-space ﬂuid (WFSF) was collected by centrifugation at 3000g at 4°C for 15min. After collecting the WFSF, the root tips were frozen at −20°C. The symplastic 1 fraction was recovered from the frozen-thawed samples by centrifugation at 3000g at 4°C for 15min. The residue was washed with 70% ethanol twice, and the combined supernatant represented the symplastic 2 fraction. The residual cell wall material was then immersed in 2M HCl for at least 24h with occasional vortexing.

The Al content in pectin was determined according to Yang *et al.* (2011). In order to avoid the chelation of Al by oxalate, cell wall material (50mg) underwent extraction for pectin twice for 1h using hot water, which showed an extraction efﬁciency similar to ammonium oxalate buffer (Yang *et al.*, 2011). The pellet (cell wall without pectin) was immersed in 2M HCl for at least 24h with occasional vortexing. The Al content of the pectin fraction was calculated by subtracting the Al content of the cell wall without pectin from the Al content of the cell wall.

The Al concentrations in the above extracts were determined on an Agilent 7500A ICP-MS (Agilent, Palo Alto, CA, USA). Al accumulation in root apexes was also detected by hematoxylin staining as described by Yamamoto *et al.* (2001).

### Determination of degree of methylation of pectin

Cell wall material from wheat root apexes (0–10mm) was prepared the same way as for pectin determination. Ten millilitres of 1M KOH were added to aliquots of the pectin fraction to give 15mL of pectin solutions. The pectin hydrolysates were neutralized with dilute H_3_PO_4_ to pH 7.5 and adjusted to 20mL with ultrapure water. Hydrolysed pectin samples (1mL) were mixed with 1mL alcohol oxidase (1 units mL^−1^) and incubated at 25°C for 15min. Then, 2mL of fluoral-P (0.02M 2,4-pentanedione in 2.0M ammonium acetate and 0.05M acetic acid) was added and vortexed. The mixtures were incubated at 60°C for 15min and then cooled to room temperature. Methanol that was released from the cell wall material was measured by fluorometry (Klavons and Bennett, 1986).

### Immunoﬂuorescence

Immunofluorescence localization of cell wall pectin was performed using specific monoclonal antibodies according to Yang *et al.* (2008). After treatment, fresh roots were cut into thin cross-sections with a freezing microtome (SLEE MTC, Germany) from root zone 1 to 3mm behind the apex, and directly ﬁxed in 4% paraformaldehyde in 50mM PIPES, 5mM MgSO_4_, and 5mM EGTA, pH 6.9. After 2h of ﬁxation at room temperature, the samples were washed repeatedly with phosphate-buffered saline (PBS, pH 7.4) and blocked with 0.2% bovine serum albumin in PBS for 30min. Then the samples were incubated for 2h with the monoclonal antibodies JIM5 (specifically labels low methylesterified pectin) and JIM7 (specifically labels high methylesterified pectin), diluted 1:10 in PBS, followed by incubation with goat anti-rat IgG (whole molecule) ﬂuorescein isothiocyanate conjugate. Finally, the samples were diluted 1:50 in PBS and left for 2h at 37°C. Samples were washed briefly with PBS three times and imaged.

### PME activity assay

For extraction of PME, root apexes (0–10mm) were homogenized and suspended in 1M NaCl solution (pH 6.0). Extracts were centrifuged at 23 000g for 10min at 4°C and the supernatant was collected. PME activity was measured according to Anthon and Barrett (2004). An incubation solution was prepared, with 100 μL of 200mM PBS containing 0.64mg mL^−1^ of pectin, 10 μL of alcohol oxidase at 0.001 units μL^−1^_,_ and 50 μL of the PME sample. Samples were incubated for 10min at 30°C, and then 200 μL of a 0.5M NaOH solution containing 5mg mL^−1^ Purpald was added. After incubation at 30°C for 30min, 550 μL of water was added to give a final volume of 1.0mL. The absorbance at 550nm was measured with a spectrophotometer (Lambda 35; PerkinElmer, Waltham, MA, USA).

### Characterization of *TaALMT1* expression

The expression of *TaALMT1* was determined by real-time quantitative reverse transcription PCR (qRT-PCR). Brieﬂy, total RNA was extracted from 100mg of fresh-weight wheat seedling root apexes (0–10mm) using Trizol reagent according to the manufacturer’s protocol (Life Technologies, Rockville, MD, USA). One microgram of total RNA from each sample was reverse-transcribed into ﬁrst-strand cDNA with a PrimeScript II 1st Strand cDNA Synthesis Kit (Takara, Dalian, Liaoning, China) according to the manufacturer’s protocol. The ﬁrst-strand cDNA was used for SYBR Green-monitored qRT-PCR (Takara). The qRT-PCR analysis was performed using the MJ Opticon™ 2 Real-Time PCR System (MJ Research). The primer pairs used for real-time PCR analysis for *TaALMT1* were those used by Tian *et al.* (2014), i.e. 5′-AAGAGCGTCCTTAATTCG-3′ and 5′-CCTTACATGATAGCTCAGGG-3′, and for the housekeeping gene *TaActin* were 5′-CTATCCTTCGTTTGGACCTT-3′ and 5′-GCGAGCTTCTCCTTTATGT-3′. The expression of *TaALMT1* was calculated from the relative expression levels of *TaALMT1* and the expression levels of the reference gene *TaActin* using arbitrary units. Three biological and three technological repeats were performed in RT-PCR. The relative expression level was analysed by the comparative C_T_ method.

### Statistical analysis

All data were statistically analysed using the SPSS package (version 11.0; SPSS Inc., Chicago, IL, USA); ANOVA was performed on the data sets, and the mean and SD of each treatment as well as least significant difference (LSD; *P* < 0.05 and *P* < 0.01) for each set of corresponding data were calculated. The figures were drawn using the software Origin 8.0 (Origin Lab Corporation, Northampton, MA, USA).

## Results

### Effect of Al and cPTIO on NO production, root elongation, and Evans Blue uptake

**Fig. 1.**
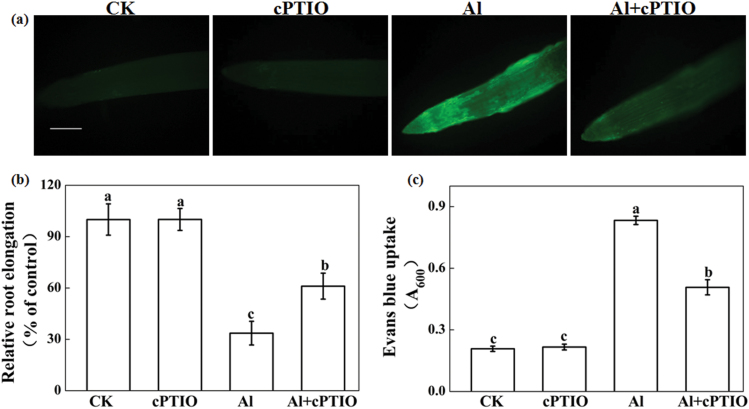
Effect of the NO scavenger cPTIO on NO content, root elongation, and Evans Blue uptake of wheat seedling roots with or without Al exposure. Three-day-old seedlings were treated with 30 μM Al and with or without 30 μM cPTIO for 24h. (**a**) Detection of NO fluorescence using DAF-FM DA staining and ﬂuorescence microscopy (n = 10). Scale bar, 1mm. (**b**) Root elongation was expressed relative to root elongation in control solutions of 0.5mM CaCl_2_, pH 4.3. Means ± SD (n = 20). (**c**) Seedling 10-mm root tips collected after 24h treatment were used to determine Evans Blue uptake. CK, 0.5mM CaCl_2_; cPTIO, 0.5mM CaCl_2_ + 30 μM cPTIO; Al, 0.5mM CaCl_2_ + 30 μM AlCl_3_; Al + cPTIO, 0.5mM CaCl_2_ + 30 μM AlCl_3_ + 30 μM cPTIO. Means ± SD (n = 3). Different letters indicate signiﬁcant differences (*P* < 0.05) among the treatments.

After treatment with 30 μM Al for 24h, a higher concentration of NO was observed in root tips of wheat (Fig. 1a), similar to that observed in our previous study (Sun *et al.*, 2014). In root tips treated with Al plus cPTIO, an NO scavenger, significantly decreased NO content was noted when compared with those treated with Al alone (Fig. 1a). Application of cPTIO significantly alleviated Al-induced root inhibition (Fig. 1b) and Evans Blue uptake (Fig. 1c). Root elongation of wheat under treatment of Al + cPTIO was 61% of the control values, but only 34% of the control values for the Al treatment alone. Similarly, cPTIO application significantly reduced Evans Blue uptake induced by Al stress. In addition, Al treatment strongly induced callose production, which is a typical indicator of Al phytotoxicity. cPTIO application significantly reduced Al-induced callose deposition (see Supplementary Fig. S1 at *JXB* online).

### Effect of cPTIO on Al accumulation in root apexes

**Fig. 2.**
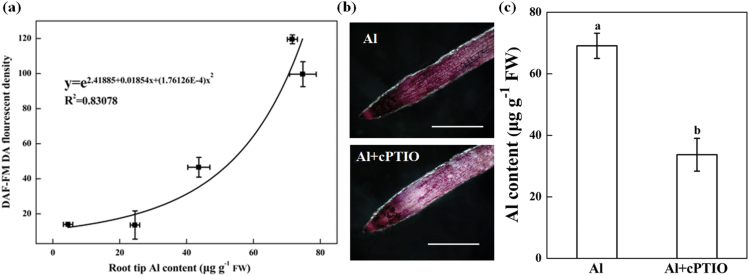
Relationship (*P* < 0.05) between Al accumulation and NO production. Three-day-old wheat seedlings were treated with 30 μM Al and with or without 30 μM cPTIO and 10-mm root tips were collected after 3, 6, 12, and 24h. (**a**) Correlation analysis of Al concentrations and NO production after 3, 6, 12, and 24h Al exposure. (**b**) Histochemical detection of Al accumulation by hematoxylin staining in the root apices after 24h of Al treatment. Scale bar, 0.5cm. (**c**) Al concentrations in root apices after 24h Al exposure.

NO generation was positively correlated with root tip Al accumulation (*P* < 0.05; Fig. 2a). cPTIO treatment of the roots in the presence of Al reduced the Al content of the root tips as demonstrated by staining of root apices with hematoxylin (Fig. 2b) and quantification of Al content (Fig. 2c).

### Effect of Al and cPTIO on malate efflux and root surface pH changes

**Fig. 3.**
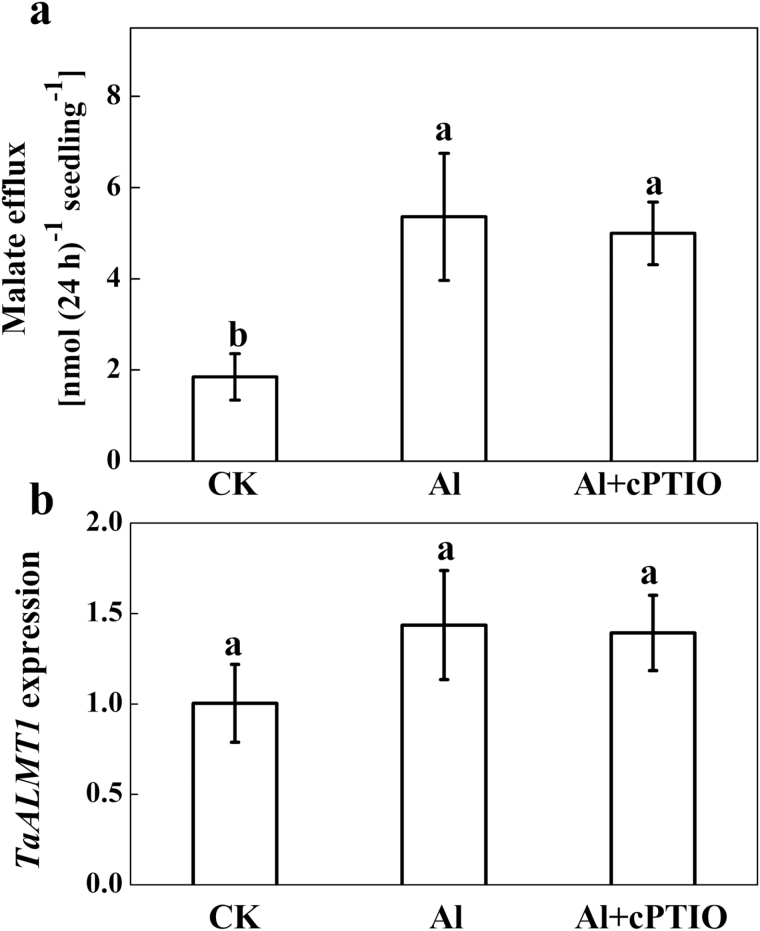
Effect of the NO scavenger cPTIO on Al-induced root malate exudation and *TaALMT1* expression in roots. Three-day-old seedlings were exposed to a 30 μM Al solution containing 0 or 30 μM cPTIO for 24h. (**a**) Root exudates were collected after 24h exposure and malate was analysed by HPLC. (**b**) Root apices (0–10mm) were collected. The relative expression of *TaALMT1*in 10-mm root apices was determined by qRT-PCR. Means ± SD (n = 3) with different letters are significantly different at *P* < 0.05.

**Fig. 4.**
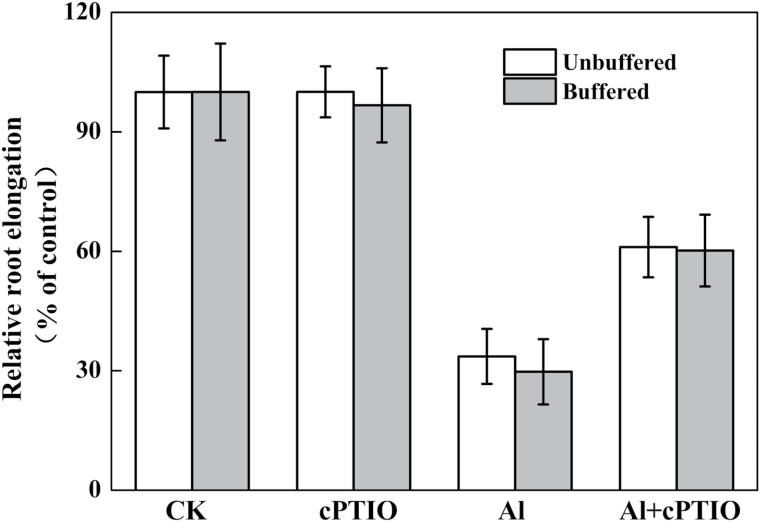
Effect of the NO scavenger cPTIO on Al-induced root growth inhibition. Three-day-old seedlings were exposed to 0.5mM CaCl_2_ solution containing 0 or 30 μM Al with or without 10mM MES in the presence or absence of 30 μM cPTIO for 24h. The pH was adjusted to 4.3. Means ± SD (n = 20).

To determine whether the decreased Al accumulation after cPTIO treatment was due to Al-induced malate secretion in wheat as demonstrated by Delhaize *et al.* (1993), malate was quantified in the root exudates (Fig. 3a). Al treatment enhanced malate efflux, but treatment with cPTIO had no effect on Al-induced malate efflux. However, the slightly, but not significantly, enhanced expression of *TaALMT1* after exposure to Al was not responsive to cPTIO application (Fig. 3b). These results indicate that the decreased Al accumulation in root apices after cPTIO treatment did not result from an increased malate efflux. Furthermore, the effect of Al and cPTIO on root elongation was independent of the buffering of the solution at pH 4.3 with MES according to Zhu *et al.* (2013) (Fig. 4).

### Effect of Al and NO scavenger on cell wall composition

**Fig. 5.**
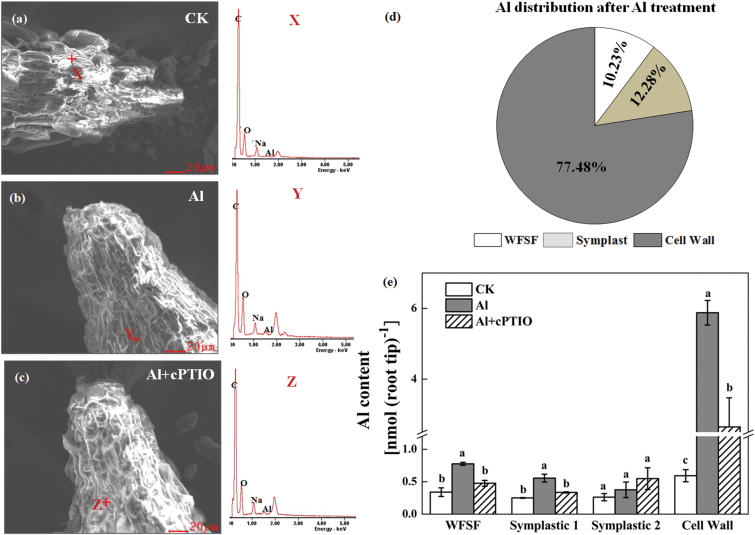
SEM-EDS spectra and Al compartmentation in wheat root apices (10mm). Representative images of SEM and EDS spectra without Al (**a**), with Al (**b**), and with Al and cPTIO treatment (**c**) are given. Relative distribution of Al (**d**) and Al contents (**e**) of cell walls, symplast, and WFSF. Wheat seedlings were exposed to 0.5mM CaCl_2_ solution containing 30 μM Al with or without 30 μM cPTIO for 24h. The relative distribution of Al was only calculated for roots treated with Al alone. Data are means ± SD (n = 3). Means with different letters are significantly different at *P* < 0.05.

Based on the SEM-EDS images shown in Fig. 5a–c, carbon and oxygen were the most abundant elements within the cell wall and Al was absorbed alongside oxygen, which indicates that Al absorption may be determined by the oxygen-containing functional groups within the surface of the root tips. Furthermore, the EDS spectrum data confirmed that cPTIO treatment significantly decreased Al content in roots of wheat under Al stress (Fig. 5b, c). Because determining the total Al content of root tips does not reveal the cellular distribution, the Al content in different fractions of the apical 1-cm root tips could not be determined (Fig. 5d, e). Under Al treatment, only a little Al was found in the symplastic fraction; however, more than 75% of the Al taken up by wheat roots accumulated in the cell wall (Fig. 5d). Application of the NO scavenger cPTIO significantly decreased cell wall Al content (Fig. 5e).

**Fig. 6.**
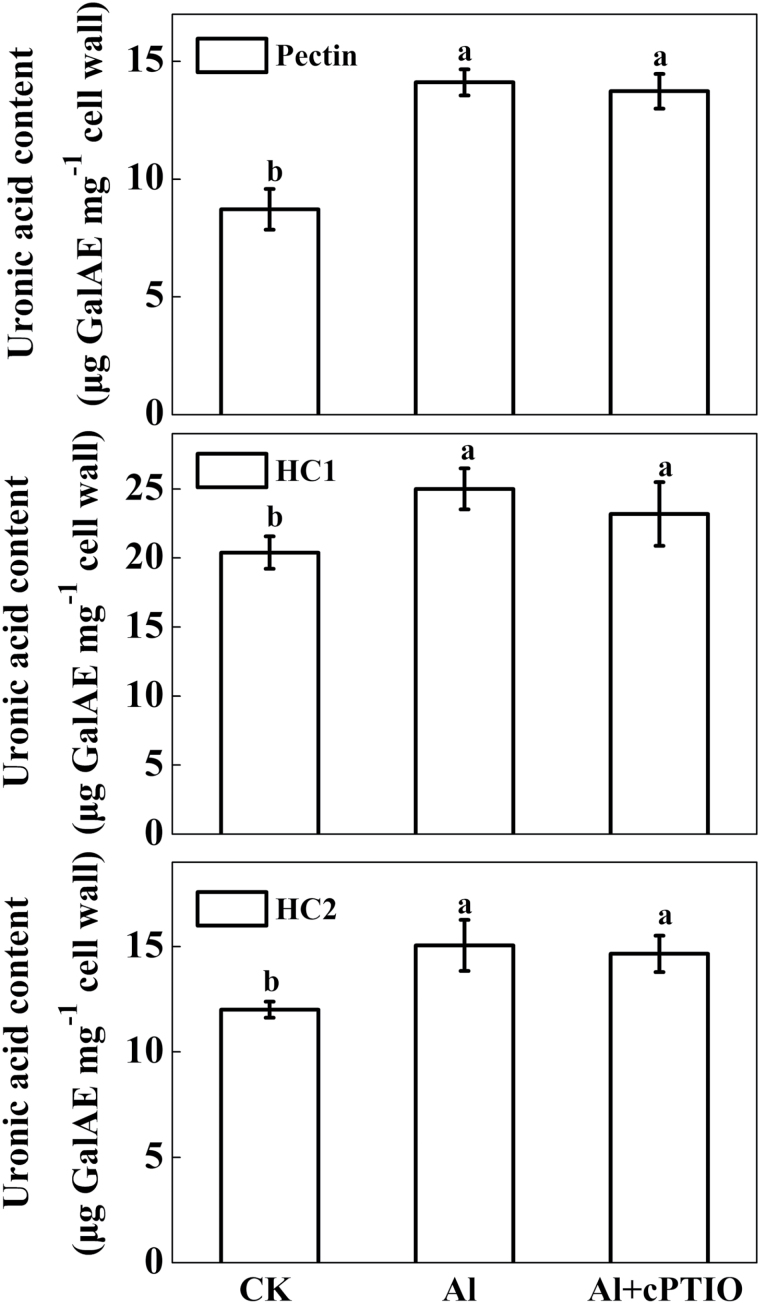
Uronic acid content of cell wall fractions extracted from the root apex of wheat. Three-day-old seedlings were exposed to 0.5mM CaCl_2_ solution containing 0 or 30 μM Al with or without 30 μM cPTIO for 24h. Cell wall polysaccharides in 10-mm root apices were fractionated into pectin, HC1, and HC2 before measurement of uronic acid content, expressed as galacturonic acid equivalents (GalAE). Means ± SD (n = 3). Bars with different letters are significantly different at *P* < 0.05.

The uronic acid content of the cell wall polysaccharides pectin, HC1, and HC2 significantly increased under Al treatment compared with the control without an Al supply, independent of treatment with the NO scavenger cPTIO (Fig. 6).

### Effect of Al and NO scavenger on pectin methylation

**Fig. 7.**
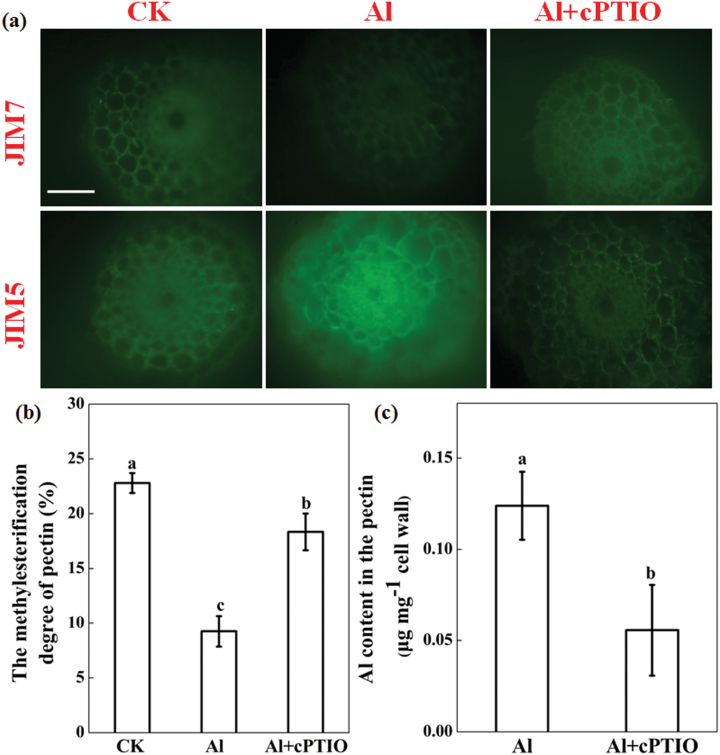
Effect of Al and cPTIO on the degree of pectin methylation and cell wall pectin Al content. Three-day-old wheat seedlings were treated with 0.5mM CaCl_2_ solution containing 0 or 30 μM Al with or without 30 μM cPTIO for 24h. (**a**) Immunolocalization of high methylesterified pectin (JIM7) and low methylesterified pectin (JIM5) in cross-sections of 10-mm root apices. Scale bars, 50 μm. (**b**) Degree of methylation of cell wall pectin extracted for root apices. (**c**) Al content of cell wall pectin. Means ± SD (n = 3). Means with different letters are significantly different at *P* < 0.05.

The monoclonal antibodies JIM5 (specifically labels low methylesterified pectin) and JIM7 (specifically labels high methylesterified pectin) were used for immunoﬂuorescence localization of cell wall pectin. As shown in Fig. 7a, Al treatment led to decreased fluorescence of JIM7, but increased fluorescence of JIM5. Contrary to Al treatment, cPTIO application increased JIM7 fluorescence and decreased JIM5 fluorescence. The degree of pectin methylation in Al-treated seedlings decreased to 40% of that without Al treatment, and treatment with cPTIO greatly restored this Al-induced decrease of pectin methylation (Fig. 7b). Depletion of endogenous NO by cPTIO strongly reduced the amount of Al in the cell wall pectin fraction compared to Al treatment alone. However, there was no difference in Al accumulation in cell wall HC1 between the wheat seedlings treated with Al and those treated with Al + cPTIO (see Supplementary Fig. S3 at *JXB* online). These results suggest that the greater Al accumulation in the cell wall could be attributed to Al-induced increased demethylation of pectin by NO.

### Effect of Al and NO scavenger on PME activity

**Fig. 8.**
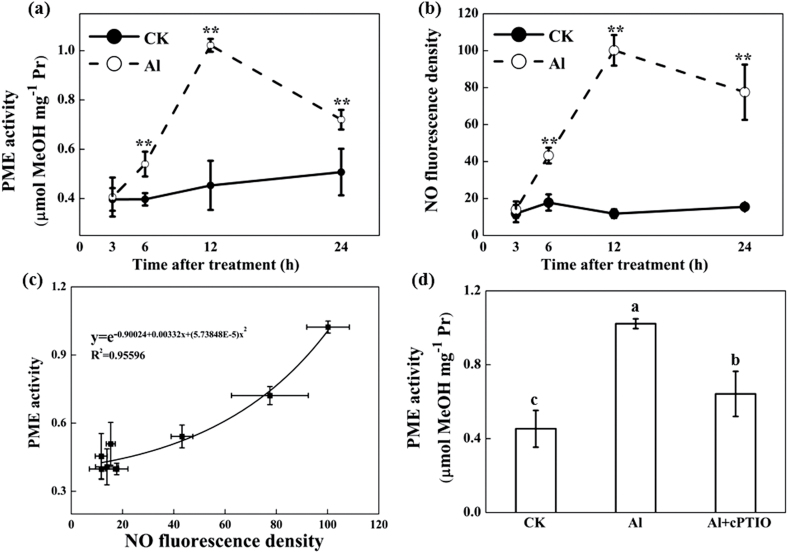
Effect of Al and cPTIO on PME activity and NO content in 10-mm root apices. Three-day-old wheat seedlings were treated with 0.5mM CaCl_2_ solution containing 0 or 30 μM Al with or without 30 μM cPTIO. PME activity (**a**), NO production (**b**), and the relationship between NO production and PME activity (**c**) after 3, 6, 12, and 24h treatment with or without Al. (**d**) PME activity after 24h treatment with and without Al and cPTIO. For quantification of NO production, root tips were loaded with 10 μM DAF-FM DA in 20mM HEPES-NaOH buffer (pH 7.4) and NO fluorescence was imaged after 20min. Images were analysed with Image-J and NO production was expressed as root fluorescence density. Means ± SD (n = 10). ** in (a) and (b) indicate significant difference between Al and control treatments at *P* < 0.01.

Al treatments resulted in a signiﬁcant increase in PME activity after 6h of treatment in comparison with no Al treatment, which peaked at 12h (Fig. 8a). A similar pattern was observed in NO production by labelling endogenous NO using DAF-FM DA (Fig. 8b). Correlation analysis suggested that NO generation was positively correlated with PME activity (Fig. 8c). cPTIO application significantly decreased PME activity induced by Al in the root tips (Fig. 8d).

## Discussion

Stress-induced NO may be endogenously produced and plays specific roles in plant responses to stress depending on the time and intensity of the NO produced (Floryszak-Wieczorek *et al.*, 2007; Puyaubert and Baudouin, 2014; Sun *et al.*, 2014). In addition to being a signalling molecule, it has been suggested that NO could also promote cytotoxic actions when produced at higher concentrations under stress conditions (Valderrama *et al.*, 2007; Besson-Bard *et al*., 2008; Leterrier *et al.*, 2012). Our previous studies suggested that an early NO burst at 3h plays an important role in Al resistance in root tips of Al-tolerant wheat genotype Jian-864 by modulating an enhanced antioxidant defence to adapt to Al stress (Sun *et al.*, 2014; Sun *et al.*, 2015), whereas the lack of NO accumulation at 3h but an extremely high NO concentration after 12h was noted in root tips of the Al-sensitive genotype Yang-5 (Sun *et al.*, 2014). The possible mechanisms involved in Al-induced high NO and its association with Al sensitivity in roots of Yang-5, however, are unknown. Results from the present study with the genotype Yang-5 show that Al significantly increased NO production and strongly impaired the root elongation and plasma membrane integrity of root tips, which was significantly reverted after treatment with the NO scavenger cPTIO (Fig. 1). These results, similar to those reported by Chen *et al.* (2014) on *Medicago sativa* (alfalfa), wheat, rice, and tomato, suggest that Al-induced high NO production contributes to Al sensitivity in root elongation in wheat.

Al accumulation in the root apex is in many cases directly related to Al toxicity (Ma, 2007; Liu *et al.*, 2014; Kochian *et al.*, 2015). Our previous study showed that the Al content of the root apex was significantly higher in the sensitive wheat genotype Yang-5 compared to the Al-tolerant genotype Jian-864 (Sun *et al.*, 2014), indicating that differing Al accumulation in the root apex might cause the variation in Al sensitivity between Yang-5 and Jian-864. In this study, we found that NO concentration was positively correlated with root tip Al accumulation. Elimination of NO by cPTIO significantly decreased Al accumulation in the root apex of wheat (Fig. 2). It is therefore possible that NO regulates Al accumulation in root tips of wheat, and subsequently increases the sensitivity of the wheat cultivar Yang-5 to Al stress. It is well documented that (i) Al-activated malate efflux (Delhaize *et al.*, 1993; Tian *et al.*, 2014) and (ii) alkalinization of the rhizosphere (Wang *et al.*, 2006) play important roles in excluding Al from wheat roots. These mechanisms, however, do not account for our finding that cPTIO prevented Al from entering wheat roots by eliminating NO. First, the Al-sensitive wheat genotype Yang-5 secreted small amounts of malate after Al exposure, and the cPTIO-treated plants secreted as much malate during the Al treatment as those treated with Al alone (Fig. 3). Wang and Yang (2005) also reported that the application of exogenous NO to the culture medium failed to induced exudation of additional organic acids as compared with Al treatment alone. Second, by buffering the solutions to pH 4.3 with MES, we excluded the involvement of alkalinization of the rhizosphere in the alleviation of Al rhizotoxicity by cPTIO (Fig. 4). These results suggest that neither malate efflux nor changes in rhizosphere pH were responsible for the reduced Al accumulation occurring after NO elimination by cPTIO.

Our investigation also suggests that decreased Al accumulation in wheat roots by cPTIO did not result from decreased cell wall polysaccharide content. The cell wall is the major site of Al accumulation and plays pivotal roles in the manifestation and perception of Al toxicity in plants (Horst *et al.*, 2010; Kochian *et al.*, 2015). It has been reported that pectic polysaccharides and hemicelluloses are the two major components to bind Al in the cell wall (Eticha *et al.*, 2005; Yang *et al.*, 2008; Horst *et al.*, 2010; Yu *et al.*, 2015). The binding of Al may change cell wall structure, making it more rigid, and reducing cell expansion and mechanical extensibility, thus inhibiting root elongation (Ma *et al.*, 2004; Kochian *et al.*, 2005; Ma, 2007; Kopittke *et al.*, 2015). In this study, we found that Al exposure induced a significant increase of uronic acid content in cell fractions, including pectin and hemicelluloses (Fig. 6). In addition, a high percentage (>70%) of the total Al accumulated by wheat roots was tightly bound to cell walls (Fig. 5d). Furthermore, a direct measurement of Al content in different cell wall components revealed that Al accumulated in the cell wall mainly in the pectin and HC1 fractions (see Supplementary Fig. S2 at *JXB* online), which was consistent with previous results found in a number of other plant species, such as wheat, maize, and rice (Tabuchi and Matsumoto, 2001; Eticha *et al.*, 2005; Yang *et al.*, 2008). It has been reported that pre-treatment of rice with exogenous NO decreased root cell wall pectin and hemicellulose content, and thus the binding of Al in root cell walls, and alleviated Al-induced inhibition of root elongation (Zhang *et al.*, 2011). However, in the present study, depletion of Al-induced endogenous NO had no effect on cell wall polysaccharide content (Fig. 6), indicating that overall content was not involved in the decreased accumulation of Al in wheat roots. It is possible that the discrepancy between the studies may be attributed to different NO concentrations, because NO affects cell wall components in a dose-dependent manner (Correa-Aragunde *et al.*, 2008).

**Fig. 9.**
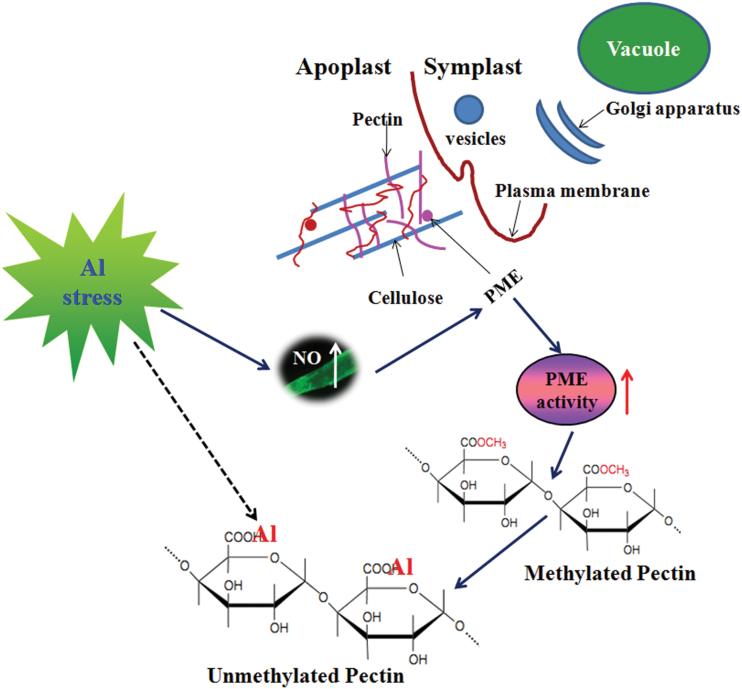
Schematic illustration of a proposed model illustrating the linkage of Al-induced NO production on pectin methylation as well as on Al binding to pectin. Al-enhanced NO production increases apoplastic PME activity, which decreases the methylation of cell wall pectin, thus increasing Al-binding capacity.

Depletion of endogenous NO acts to decrease Al accumulation in the cell wall, mainly by modulating the enhanced methylation of pectin. Pectic polysaccharides and hemicelluloses, in particular xyloglucan, in the walls are the two major components to bind Al in the wall (Zhu *et al.*, 2014). Depletion of Al-induced endogenous NO by cPTIO significantly decreased pectin Al accumulation but had no effect on Al accumulation in HC1 (see Supplementary Fig. S3 at JXB online), indicating that endogenous NO affects the Al-binding capacity of the cell wall, probably by interfering with pectin structure but not HC1. Pectins are secreted into the wall as highly methylated and then undergo partial apoplastic demethylation processes through the action of PME, resulting in the exposure of free pectic carboxylic groups, which could serve as binding sites for Al in the cell wall (Zhu *et al.*, 2014). Schmohl and Horst (2000) reported that the Al sensitivity of maize cell suspension cultures was negatively related to the degree of pectin methylation. In the present study, Al exposure significantly increased PME activity (Fig. 8a). Using immunofluorescence localization of pectin with two types of antibodies, the spatial distribution of both low-methylesterified and high-methylesterified pectin was determined. The low-methylesterified pectin was significantly enhanced in the epidermis and vascular tissues in Yang-5 root tips after Al exposure (Fig. 7a), and this was consistent with previous observations on an Al-sensitive rice cultivar (Yang *et al.*, 2008). These results support the hypothesis that Al decreases the methylation level of pectin, and consequently results in higher Al binding in the cell wall, which is in agreement with results reported in maize and rice (Eticha *et al.*, 2005; Yang *et al.*, 2008). However, visualization of cells under the microscope indicated a much higher intensity of low-methylesterified pectin in the vascular regions after Al exposure as compared with the controls, although in Al excluders like wheat Al is not readily transported into the central cylinder. This could be due to the systemic effect of Al on NO formation. In agreement with the immunofluorescence localization of pectin, depletion of endogenous NO resulted in significant decreases in PME activity (Fig. 8c), leading to a significantly increased degree of methylation of pectin and less Al binding in pectin (Fig. 7). These results suggest that the decreased accumulation of Al in root tips after cPTIO treatment was the result of a decreased degree of cell wall pectin demethylation. Results from reinforcing NO production in the root tips of *Vigna umbellata* (rice bean) under Al stress provided additional evidence that increased NO enhances PME activity and pectin demethylation, and ultimately increases the accumulation of Al in cell wall (Zhou *et al.*, 2012). Based on the results of our study, we propose a model to illustrate the linkage of Al-induced NO on pectin methylation as well as to Al sensitivity (Fig. 9). Al-induced increased endogenous NO accumulation removes methyl groups from pectin by activating PME, and causes enhanced Al binding to unmethylated carboxyl groups that have a high afﬁnity for Al.

In summary, our study reveals a negative role of Al-induced NO production in response to Al stress in roots of the Al-sensitive *T. aestivum* genotype Yang-5. The mechanistic basis of the process is presumably through increased NO-regulated PME activity and thus decreased pectin methylation of the cell wall. Consequently, Al is able to target unmethylated pectin more easily and more Al is bound in the cell walls in this plant species.

## Supplementary data

Supplementary data are available at *JXB* online.

Fig. S1. Effects of cPTIO on the Al-induced callose production in root apexes of Yang-5.

Fig. S2. Al contents in different cell wall components.

Fig. S3. Effect of cPTIO on Al accumulation of different cell wall polysaccharides in roots of Yang-5.

Supplementary Data

## Abbreviations:

Al,: Aluminium;
NO,: Nitric oxide;
cPTIO,: 2-(4-carboxyphenyl)-4,4,5,5-tetramethylimidazoline-1-oxyl-3-oxide;
DAF-FM DA,: Diaminoﬂuorescein-FM diacetate;
PME,: Pectin methylesterase;
HC1,: Hemicellulose 1;
HC2,: Hemicellulose 2;
GalA,: Galacturonic acid.
