# Supplementary material for: Decreasing methylation of pectin caused by nitric oxide leads to higher aluminium binding in cell walls and greater aluminium sensitivity of wheat roots
Source: J Exp Bot. 2015 Dec 9;67(3):979–89. doi: 10.1093/jxb/erv514 (PMC4737084; doi:10.1093/jxb/erv514)
Supplement: Supplementary Data [file supp_67_3_979__index.html]

Decreasing methylation of pectin caused by nitric oxide leads to higher aluminium binding in cell walls and greater aluminium sensitivity of wheat roots — Decreasing methylation of pectin caused by nitric oxide leads to higher aluminium binding in cell walls and greater aluminium sensitivity of wheat roots — Supplementary Data 

# Decreasing methylation of pectin caused by nitric oxide leads to higher aluminium binding in cell walls and greater aluminium sensitivity of wheat roots

## Supplementary Data

Data files

- Supplementary\_figures\_S1\_S3.pdf - Supplementary Data
